# Effect of Glycosylation on Self-Assembly of Lipid A Lipopolysaccharides in Aqueous Solutions

**DOI:** 10.1021/acs.langmuir.3c00828

**Published:** 2023-06-08

**Authors:** Valeria Castelletto, Jani Seitsonen, Ian W. Hamley

**Affiliations:** † School of Chemistry, Food Biosciences and Pharmacy, University of Reading, Whiteknights, Reading RG6 6AD, U.K.; ‡ Nanomicroscopy Center, Aalto University, Puumiehenkuja 2, Espoo FIN-02150, Finland

## Abstract

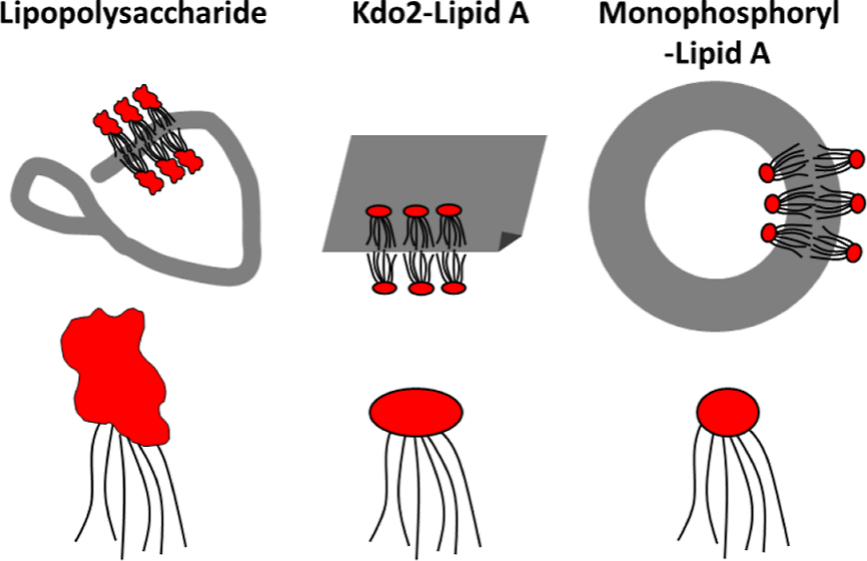

Lipopolysaccharides (LPSs) based on lipid A produced by bacteria are of interest due to their bioactivity in stimulating immune responses, as are simpler synthetic components or analogues. Here, the self-assembly in water of two monodisperse lipid A derivatives based on simplified bacterial LPS structures is examined and compared to that of a native *Escherichia coli* LPS using small-angle X-ray scattering and cryogenic transmission electron microscopy. The critical aggregation concentration is obtained from fluorescence probe experiments, and conformation is probed using circular dichroism spectroscopy. The *E. coli* LPS is found to form wormlike micelles, whereas the synthetic analogues bearing six lipid chains and with four or two saccharide head groups (Kdo_2_-lipid A and monophosphoryl lipid A) self-assemble into nanosheets or vesicles, respectively. These observations are rationalized by considering the surfactant packing parameter.

## Introduction

Glycosylated polymers and oligomers can undergo self-assembly driven by amphiphilicity, *i.e.*, the presence of both hydrophilic and hydrophobic parts. This in turn may influence bioactivity. Recently, lipopolysaccharides (LPSs) with low dispersity have been developed, and here, we examine the self-assembly of two such model systems in comparison with native polydisperse bacterial LPS.

**Scheme 1:**
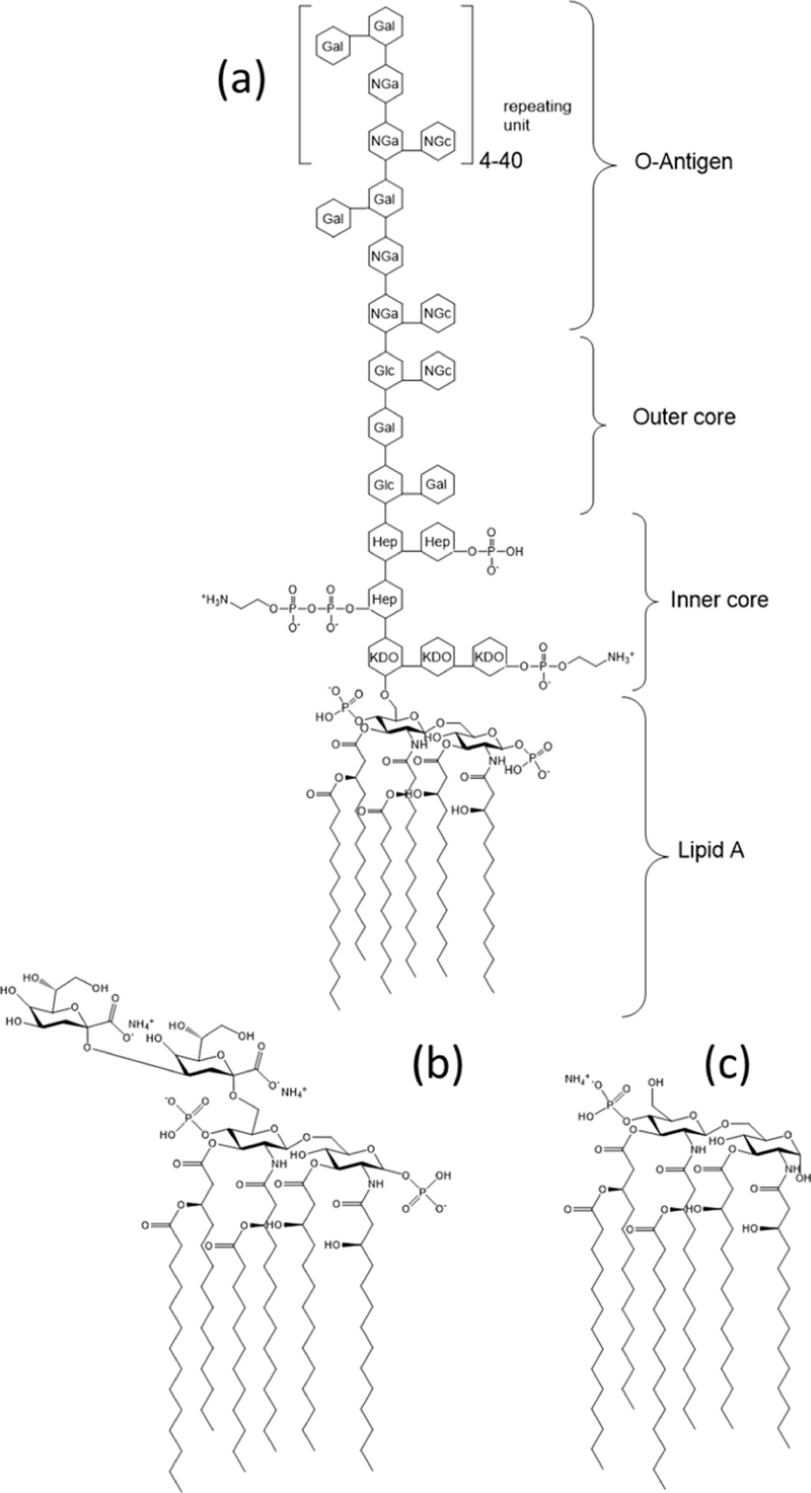
(a) Schematic of LPS from *E. coli* (Adapted from Refs (14) and (15)), (b) Kdo_2_-Lipid A (Kdo2L), and (c) MPLA Abbreviations: KDO: 3-deoxy-α-d-*manno*-octulosonic acid; Hep: heptulose (ketoheptose); NGa: galactosamine; NGc: glucosamine.

The recent COVID-19 pandemic has focused attention on vaccine adjuvants, which boost the immunogenic response of vaccines. A range of lipid-based adjuvants have been developed and formulated of which those based on, or inspired, by bacterial LPSs are one class expected to have good activity in stimulating immune response. The outer membrane of Gram-negative bacteria such as *Escherichia coli* has an asymmetric structure; the inner leaflet is a phospholipid bilayer, and the outer leaflet is LPS. Bacterial LPSs vary from species to species and have considerable dispersity in the number and type of constituent sugars, and there has thus been considerable interest in nontoxic, simplified (and less disperse) immunogenic components such as monophosphoryl lipid A (MPLA)^1−3^ and Kdo_2_-lipid A (Kdo2L).^4−6^ These both contain lipid A, which contains multiple acyl chains (often six in a highly immunogenic form) with typically 10–16 carbon atoms (Scheme 1). MPLA has a structure based on a disaccharide of glucosamide decorated with six C_14_ lipid chains and is *mono*phosphorylated (Scheme 1). MPLA was developed since its high purity means it can be used to selectively activate TLR4, and it has widely been explored as a vaccine adjuvant.^1,2,7−13^ Kdo2L comprises two units of the saccharide Kdo (3-deoxy-α-d-*manno*-octulosonic acid) linked to lipid A. It is a core component of LPS in Gram-negative bacteria which comprises a minimal unit for bacterial viability, and like MPLA, it also activates TLR4.^4−6^

LPS is a polydisperse component of the membranes of different bacteria. A representative structure for an *E. coli* LPS is shown in Scheme 1. It contains the lipid A structure (diphosphorylated, *i.e.*, with two negatively charged groups) in which the lipid chain length and the number of acyl groups depend on the bacterial strain, although these are relatively conserved within a species.^16,17^ Saccharides are linked to the lipids in the inner core, and there are further outer core and O-antigen regions. The inner polysaccharide core typically contains between one and four units of Kdo, which is specifically associated with LPS.^16,18^ The Kdo-containing inner core is also modified with heptulose (ketoheptose) monosaccharides, the most common of which is l-*glycero*-α-d-*manno*-heptopyranose. The inner core glycan residues are typically phosphorylated or modified with phosphate-containing groups, *e.g.*, pyrophosphate or 2-aminoethylphosphate. The outer core of the LPS contains more common hexoses, such as glucose, galactose, and *N*-acetylglucosamine, and is structurally more diverse than the inner core.^16,17^ The O-antigen is an oligosaccharide unit typically comprising 1–8 glycosyl residues (5 are shown in the structure in Scheme 1).^16^ The O-antigen is the main component of LPS that differentiates bacteria. The structures and functions of different types of LPSs have been reviewed.^16^

A key question is whether LPS and related monodisperse derivatives exert their bioactivity in aggregated or unaggregated forms.^19^ Studies indicate that LPS dissociates from an aggregated form in the initial step in the activation of responding cells by LPS.^20,21^ In contrast, Mueller *et al.* reported that a rough mutant LPS and lipid A are only active in the aggregated form.^22^

Free lipid A extracted from different Gram-negative bacteria shows lamellar or nonlamellar (cubic or hexagonal) structures at high concentrations in water.^23^ Under similar conditions (solutions with a 15% lipid content), Kdo2L (from *Neisseria meningitidis*) also shows a nonlamellar (probable cubic) structure, in contrast to the LPS from this bacterium, which forms unilamellar structures.^24^ It should be noted that these small-angle X-ray scattering (SAXS) studies employed concentrations far in excess of those *in vivo*. Kdo2L (from *E. coli*,^4^ now commercially available) aggregates above a critical aggregation concentration (CAC);^19^ however, the structure of the aggregate was not determined. Dynamic light scattering was used to measure the size of preaggregate oligomers, and differential scanning calorimetry was used to infer the presence of a gel–liquid crystal-phase transition of lamellae.^19^ Cryogenic transmission electron microscopy (cryo-TEM) reveals branched and looped aggregates in Tris buffer solutions of *E. coli* LPS,^25^ while small-angle neutron scattering (SANS) and cryo-TEM on the same LPS in a MgCl_2_ solution (or mixtures with antimicrobial peptide LL37) also reveal branched “wormlike” micelle structures in the LPS solutions.^26^

Here, we compare the aggregation behavior in water of MPLA and Kdo_2_-lipid A with that of LPS using fluorescence probe measurements to determine CAC values and circular dichroism (CD) spectroscopy to probe the conformation. Cryo-TEM and SAXS are combined to examine and compare in detail for the first time the self-assembled nanostructures of the three systems. Distinct self-assembly behaviors are revealed using a powerful combination of cryo-TEM and SAXS.

## Experimental Section

### Materials

MPLA was obtained from Invivogen (Toulouse, France). MPLA is synthetic monophosphoryl lipid A with six fatty acyl groups. It is structurally very similar to natural MPLA except that natural MPLA contains a mixture of 5, 6, and 7 acyl lipid A, whereas synthetic MPLA is monodisperse with a molar mass of 1763.47 g mol^–1^. Kdo_2_-lipid A (Kdo2L) (purity > 95%, ammonium salt) was obtained from Sigma-Aldrich. It also bears six lipid chains and has molar mass 2306.84 g mol^-1^. LPS denotes a mixture of LPSs from *E. coli* O111:B4, purified by phenol extraction, obtained from Sigma-Aldrich. The structures of the three LPSs studied are shown in Scheme 1.

### Fluorescence Spectroscopy

Experiments were carried out using a Varian Model Cary Eclipse spectrofluorometer. The determination of the CAC value was estimated by titration of the fluorescent dye pyrene. The fluorescence of pyrene was excited at 335 nm at room temperature, and emission spectra were recorded from 350 to 450 nm. Excitation and emission bandwidths of 5 nm were used throughout the experiments. LPS solutions were loaded in a 10 mm-path-length quartz cell, while Kdo2L and MPLA solutions were loaded in a 3 mm-path-length quartz cell.

For experiments with LPS and Kdo2L, an initial mother solution was prepared, containing 0.05 wt % liposaccharide dissolved in a 1.3 × 10^–5^ M solution of pyrene in water. The mother solution was then used to prepare each peptide solution in the 1.3 × 10^–5^ M pyrene dilution series.

For MPLA, a mother solution of 0.05 wt % MPLA in 1.3 × 10^–5^ M of pyrene in water was initially prepared, but the solubility of the MPLA had to be improved by adding dimethyl sulfoxide (DMSO) such that the final conditions for the mother solution were 0.05 wt % MPLA in 1.3 × 10^–5^ M pyrene in 99.4% water/0.6% DMSO. The sequence of dilutions were prepared from the mother solution by dissolving it in 1.3 × 10^–5^ M pyrene in 99.4% water/0.6% DMSO.

### CD Spectroscopy

CD spectra were recorded using a Chirascan spectropolarimeter (Applied Photo Physics, Leatherhead, UK). Solutions were placed between parallel plates (0.1 mm path length). Spectra were measured with a 0.5 nm step, a 0.5 nm bandwidth, and a 1 s collection time per step. The CD signal from the water background was subtracted from the CD data of the sample solutions. The CD signal was smoothed using the Chirascan software for data analysis. The residue of the calculation was chosen to oscillate around the average to avoid artifacts in the smoothed curve.

LPS and Kdo2L samples were dissolved in water. MPLA was first dissolved in 1,1,1,3,3,3-hexafluoro-2-propanol (HFIP), and then water was added to the solution to give 0.4 wt % MPLA in 75% water/25% HFIP. A small open vial containing the HFIP/water MPLA solution was placed on a hot plate. The solution was stirred while heated at 60 °C (58.2 °C: boiling point of HFIP). The volume of the solvent was reduced to give a 0.9 wt % MPLA solution at pH 7. From the final volume of the solution and the pH value, it was estimated that following evaporation at 60 °C, the sample composition was 0.9 wt % MPLA in water.

The same protocols were followed to prepare the solutions for cryo-TEM and SAXS described below.

### Cryogenic-TEM

Imaging was carried out using a field emission cryoelectron microscope (JEOL JEM-3200FSC), operating at 200 kV. Images were taken in bright field mode and using zero loss energy filtering (omega type) with a slit width of 20 eV. Micrographs were recorded using a Gatan UltraScan 4000 charge-coupled device camera. The specimen temperature was maintained at −187 °C during the imaging. Vitrified specimens were prepared using an automated FEI Vitrobot device using Quantifoil 3.5/1 holey carbon copper grids with a hole size of 3.5 μm. Just prior to use, grids were plasma-cleaned using a Gatan Solarus 9500 plasma cleaner and then transferred into the environmental chamber of an FEI Vitrobot at room temperature and a 100% humidity. Thereafter, 3 μL of the sample solution was applied on the grid and it was blotted twice for 5 s and then vitrified in a 1/1 mixture of liquid ethane and propane at a temperature of −180 °C. The grids with the vitrified sample solution were maintained at liquid nitrogen temperature and then cryotransferred to the microscope.

### Small-Angle X-ray Scattering

Synchrotron SAXS experiments on solutions were performed using BioSAXS robots on beamline B21 at Diamond (Didcot, UK)^27^ or BM29 at the ESRF (Grenoble, France).^28^ A few microliters of each sample were injected *via* an automated sample exchanger at a slow and very reproducible rate into a quartz capillary (1.8 mm internal diameter) in the X-ray beam. The quartz capillary was enclosed in a vacuum chamber in order to avoid parasitic scattering. After the sample was injected into the capillary and reached the X-ray beam, the flow was stopped during the SAXS data acquisition. At Diamond, the *q* range was 0.007–0.33 Å^–1^, with a wavelength λ = 1.03 Å. The images were captured using a Pilatus 1M detector. At the ESRF, the *q* range was 0.005–0.48 Å^–1^, with λ = 0.99 Å, and the images were obtained using a PILATUS 3-2M detector. Data processing (background subtraction and radial averaging) was performed using dedicated beamline software.

## Results and Discussion

**Figure 1:**
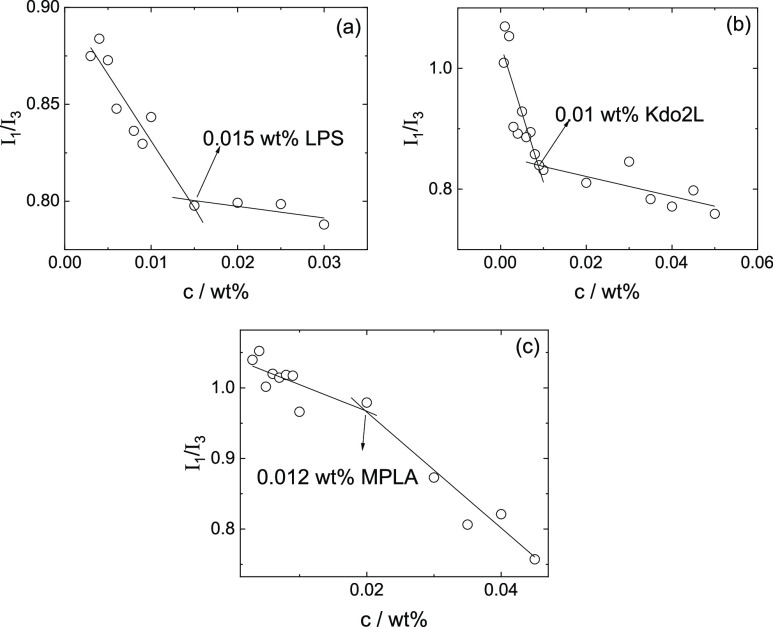
Pyrene fluorescence assay of CAC using the *I*_1_/*I*_3_ vibronic band intensity ratio for (a) LPS, (b) Kdo2L, and (c) MPLA.

The possible existence of CACs for the three lipid A poly- (oligo-) saccharides was studied using pyrene fluorescence probe assays *via* analysis of the vibronic band *I*_1_/*I*_3_ ratio, which is sensitive to the local hydrophobic environment.^29,30^ The fluorescence intensity of the first vibronic band (λ = 373 nm) is denoted *I*_1_, and *I*_3_ (λ = 383 nm) is the fluorescence intensity of the third principal vibronic band.^29,31^ The original spectra are shown in Supporting Information Figure S1. The pyrene fluorescence assay data in Figure 1a for LPS shows the expected decrease^29,31^ in *I*_1_/*I*_3_ with increasing concentration with a discontinuity in *I*_1_/*I*_3_ at 0.015 wt %, which indicates the CAC. For comparison, the CAC of *E. coli* 0111:B4 LPS was previously reported on the basis of an *N*-phenyl-1-naphthylamine fluorescence assay to be 0.0022 wt % in a Tris/NaCl buffer (pH 7.5).^18^ This significantly lower value presumably reflects charge screening in the buffer solution which facilitates aggregation at lower concentrations than in water. For Kdo2L, the concentration dependence of *I*_1_/*I*_3_ in Figure 1b shows a break at 0.01 wt %, identified as the CAC. For MPLA, the concentration dependence of *I*_1_/*I*_3_ in Figure 1c shows a break at 0.012 wt %, identified as the CAC. It was also observed that there is a discontinuity in the pyrene *I*_1_ band intensity at the CAC for both MPLA and LPS at a similar concentration to that at which breaks are observed in *I*_1_/*I*_3_. This phenomenon was previously reported and discussed by us when investigating the CAC of a peptide amphiphile.^32^

The conformation of the LPSs was investigated using CD spectroscopy. The spectra are shown in Supporting Information Figure S2. LPS similar to our sample [*i.e.*, from *E. coli* (0111:B4)] in Tris buffer has previously been reported to have a CD spectrum with a broad negative CD band in the range of 200–240 nm.^25,33^ CD spectra for LPS from *Salmonella minnesota* (prepared as hydrated multilayers) show a negative CD peak at around 190 nm and a broad negative minimum at around 220 nm.^34^ Our measured spectra are qualitatively similar to these prior reports, although, at the higher concentration studied (0.5 wt %), there are notable negative bands (Cotton effect, presumably of C=O chromophores) at 207, 222, and 243 nm pointing to the chiral superstructure, potentially with a right-handed twist. To the best of our knowledge, CD spectra have not previously been reported for monodisperse lipid A derivatives. The CD spectra obtained for Kdo2L (Supporting Information Figure S2b) are qualitatively similar to those for LPS at the same concentration (data presented for 0.1 and 0.5 wt % solutions). This indicates that the substantial reduction of the polysaccharide moiety (Scheme 1) does not influence the local chiral environment. In contrast, further simplification to the disaccharide head group in MPLA leads to a CD spectrum (Supporting Information Figure S2c) characteristic of a disordered structure, being largely featureless other than a large minimum near 195 nm.

**Figure 2:**
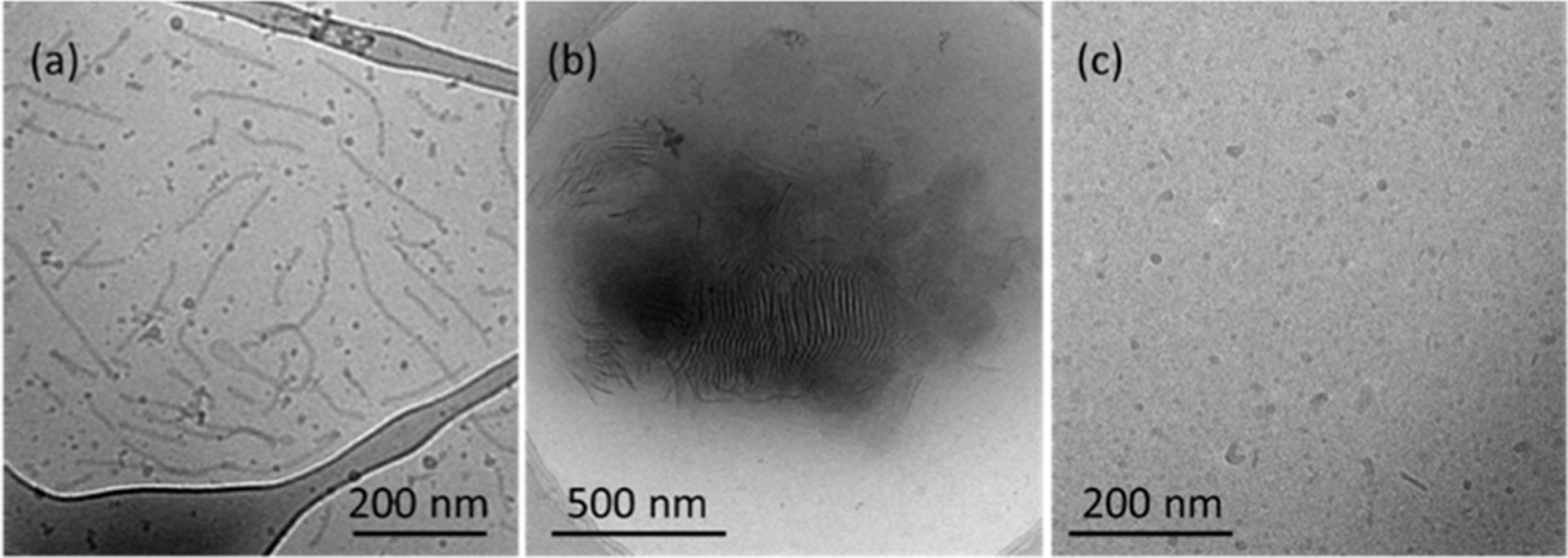
Cryo-TEM images from 0.5 wt % solutions of (a) LPS, (b) Kdo2L, and (c) MPLA.

Having established that the three LPSs aggregate at sufficiently high concentration, we used detailed cryo-TEM and SAXS measurements to comprehensively characterize the nature, internal structure, and dimensions of self-assembled nanostructures above the CAC, through a combination of real-space high-resolution imaging on vitrified samples and *in situ* scattering methodology. Cryo-TEM images are shown in Figure 2 (additional images are provided in Supporting Information Figures S3–S5). LPS shows elongated and branched micelles with occasional toroids (Figure 2a). Similar cryo-TEM images have previously been presented for *E. coli* 0111:B4 LPS in Tris buffer,^25^ or in a 1 mM MgCl_2_ solution,^26^ although here, in aqueous solutions, coexistence of elongated structures with globular structures resembling vesicles is observed. Figure 2b shows a cryo-TEM image for Kdo2L which shows irregular nanosheet structures which are stacked on top of one another in some areas. Remarkably, changing from a tetrasaccharide unit in Kdo2L to a disaccharide head group in MPLA leads to a very distinct mode of self-assembly. For MPLA, cryo-TEM reveals the presence of vesicles (Figure 2c) with a wide distribution of sizes (up to a few tens of nanometers in diameter).

**Figure 3:**
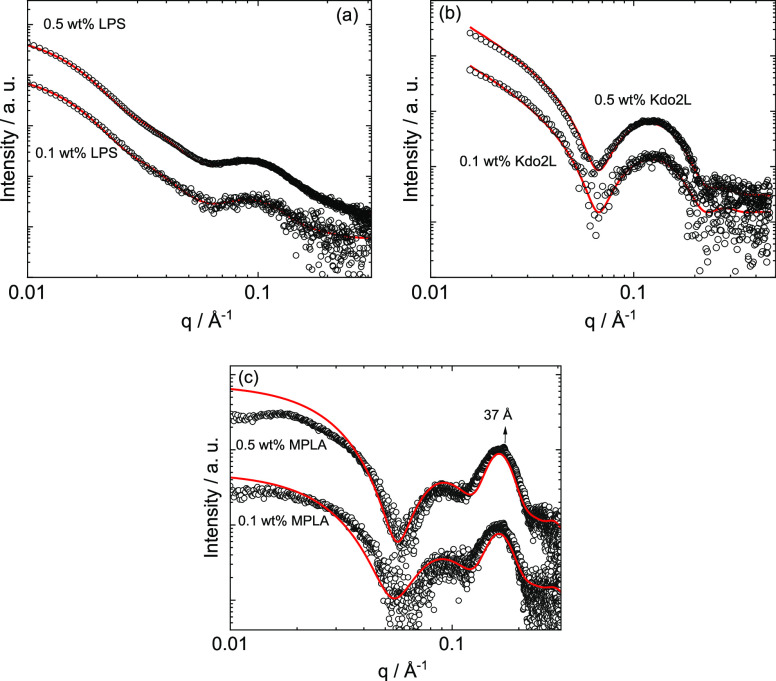
SAXS data (black symbols) with model fits (solid red lines) described in the text, (a) LPS, (b) Kdo2L, and (c) MPLA (for ease of visualization, only every fifth data point is plotted in parts a and c and every second data point in part b).

The SAXS data for the three LPSs are shown in Figure 3, and they clearly show substantial differences comparing samples. The SAXS data for LPS was fitted with a model consistent with the structures observed in the cryo-TEM image (Figure 3a), *i.e.*, elongated cylindrical micelles along with globular (pseudospherical) objects. The model form factor used was a combination of that for a long cylindrical shell structure and that for a uniform sphere. Excellent fits were obtained using this model. The fit parameters are listed in Table S1 and indicate that the cylindrical micelle fraction has a radius of 19.3 ± 6.6 Å with a 20 Å thick shell, whereas the globular spherical structures have an average outer radius of 124 Å, although there is considerable polydispersity (Gaussian standard deviation σ = 40 Å) consistent with the size polydispersity in the cryo-TEM images (*e.g.*, Figure 2). These values may be compared with those obtained from SANS by Bello *et al.* in their study of *E. coli* LPS in a MgCl_2_ solution and mixtures with antimicrobial peptide LL37.^26^ They found, from a Kratky plot analysis,^35^ a cross-section radius (, where *R*_g_ is the cross-section radius of gyration) of wormlike micelles of 35–37 Å for LPS 0111:B4 (for a range of concentrations), *i.e.*, *R*_g_ = 24–26 Å. Fitting using a flexible cylinder model for LPS D21 (a strain mutant) provides lower radii of 21–13 Å depending on the peptide content (the higher value is for an LPS/LL37 ratio of 50:1). This is very close to the core radius *R*_c_ = 19.3 ± 6.6 Å obtained from our fits, which is also close to the above *R*_g_ values from Kratky plot analyses.

Consistent with the cryo-TEM image showing nanosheets, the SAXS data for Kdo2L (Figure 3b) were fitted to a form factor model for planar structures with a graded electron density profile perpendicular to the sheet surface, represented as a sum of three Gaussian functions, two for the outer surfaces and one for the inner hydrophobic core (with negative electron density). This model is termed the Gaussian bilayer model,^36^ originally used for lipid bilayers and recently employed by our group to represent the form factor of various peptide and lipopeptide nanosheets and nanotape assemblies.^37,38^ This model fits the data very well (Figure 3b), and the fit parameters are listed in Supporting Information Table S2. The total thickness of the nanosheets is *t* = 42 Å, which is consistent with a bilayer structure considering the estimated molecular length (*ca.* 30 Å). The SAXS data for MPLA (Figure 3c) can be fitted using the form factor of a bilayer vesicle, consistent with the cryo-TEM image in Figure 2c. An additional structure factor term was necessary to account for the peak corresponding to the layer repeat distance *d* = 37 Å. The model describes the data well (although there are additional structure factor effects at low *q* for the higher concentration) and indicates that the structure comprises multilamellar vesicles (with approximately two bilayers) with a small core radius. The data at the two concentrations studied superpose, other than a scale factor and different background. The fit parameters are listed in Table S3 and show that the vesicles have a small core (radius 5 Å) with a head group sublayer thickness of 14.5 Å and a tail sublayer thickness of 13 Å. These are physically reasonable considering the likely size of the saccharide head group and C_14_ tails (Scheme S1). The total vesicle diameter from the SAXS fit is 94 Å, which is reasonable considering the cryo-TEM image in Figure 2c which may also show clusters of vesicles or irregular globular aggregates, as well as the main population of small vesicles revealed by SAXS.

**Figure 4:**
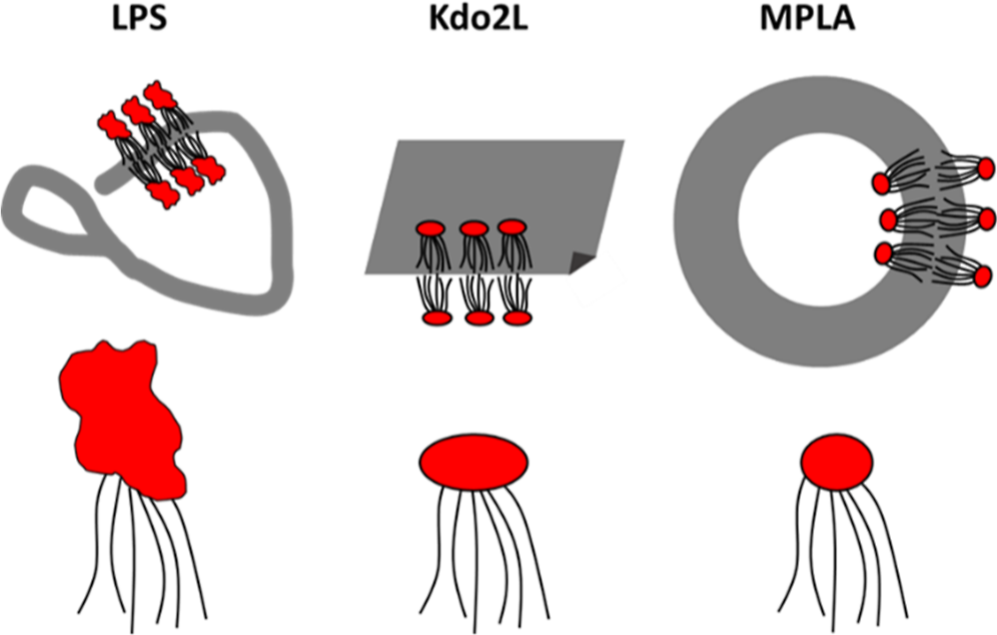
Schematic of self-assembled structures observed for LPS (branched wormlike micelles), Kdo2L (nanosheets), and MPLA (vesicles) with cartoon molecular structures.

The combination of cryo-TEM and SAXS provides clear information on the nanostructures formed by the three lipid A LPSs. This leads to the proposed models shown in Figure 4. LPS forms branched wormlike micelles (coexisting with globular structures), and the positive curvature structures presumably reflect the asymmetry of the molecular structure which comprises a very bulky (and polydisperse) saccharide “headgroup” with a large cross-section even compared to the six lipid chains in the molecule. In contrast, Kdo2L comprises a much smaller and monodisperse tetrasaccharide hydrophilic unit attached to the lipid chains. This leads to a balanced interfacial area and the formation of planar nanosheet structures. Finally, further simplification of the molecular structure in MPLA with a disaccharide head group leads to self-assembly into vesicles based also on an LPS bilayer as for Kdo2L but with an additional tendency for membrane spontaneous curvature. It is possible that curvature may be present in the bilayers of MPLA but not Kdo2L due to the lower charge on the former compared to the latter which could result in reduced membrane rigidity for MPLA bilayers; there is also the possibility for self-sorting of molecules between the inner and outer leaflet in the MPLA vesicles due to differences in the charge state or molecular structure.

The formation of specific nanostructures by amphiphilic molecules can be rationalized using the surfactant packing parameter model, based on a simple geometric analysis of molecular packing into different-shaped aggregates.^39^ We estimated the surfactant packing parameter *p* = *v*/*al*, where *v* is the volume of the hydrophobic region, *a* is the effective area per head group, and *l* is the length of the hydrophobic unit, for the three glycolipids studied. For Kdo2L, X-ray reflectivity data on Langmuir monolayers^40^ provides values for *a* = 125 Å^2^ and *l* = 13.1 Å. The volume of the hydrophobic moiety can be estimated using the equation due to Tanford for the volume per lipid chain,^41^*v*/Å^3^ = 27.4 + 26.9*n* (where *n* is the number of carbons in the lipid chain; here, *n* = 14 for five chains and *n* = 12 for one chain since Kdo2L comprises five C_14_ chains and one C_12_ chain), as previously employed for synthetic lipid A liposaccharides.^42^ This yields *v* = 2370 Å^3^ for Kdo2L and thus *p* = 1.4, which is reasonably close to *p* ≈ 1 expected for lamellar structures,^39,43^ considering the approximations involved in this estimation. In fact, an alternative estimate can be obtained using the Tanford equation for the molecular length,^41,42^*l*/Å = 1.54 + 1.265*n*, rather than that obtained by modeling X-ray reflectivity (for monolayers at the air–water interface). This equation yields *l* = 19.3 Å and thus *p* = 1.0, exactly as expected. For MPLA, surface pressure–area isotherm Langmuir trough measurements on spread monolayers provide *a* = 119 Å^2^ in the condensed state.^44^ MPLA has almost the same lipid chain structure as Kdo2L, only differing in that it contains six C_16_ chains, giving *v* = 2424 Å^3^ and assuming *l* = 19.3 Å using the Tanford formulae as for Kdo2L, which gives *p* = 1.1. As a note on the accuracy of the Tanford approximation of molecular volume, the estimated molecular volume from the Langmuir trough compression measurements was reported as *v* ≈ 2600 Å^3^,^44^ which is within 10% of the calculated value. For LPS, we approximate *v* and *l* with the values for Kdo2L and MPLA since the hydrophobic portion of the molecule is similar (Scheme 1), although with greater dispersity in lipid chain number and length. The parameter that is changed substantially compared to these synthetic liposaccharides is the effective area per head group, which is significantly larger for LPS (Scheme 1). This quantity can be estimated. There are a large number of reported studies on monolayer films from X-ray reflectivity and surface pressure–area measurements among other methods for LPS from different bacteria, as reviewed recently.^45^ Typical values are in the range *a* = 140–300 Å^2^ for the condensed phase of monolayers.^46−54^ Using *a* = 300 Å^2^ (this is an upper estimate from the range of values in the literature; however, it is reported for an *E. coli* wild-type LPS strain^54^), we estimate *p* = 0.4 which is within the range of  expected for cylindrical micelles.^39,43^ However, clearly, the estimation is subject to considerable uncertainty in not just the value of *a* but also *v* and *l*(47−49,51,53−56) for different LPS variants. This arises due to factors including differences in quantities measured using various methods (and their precise definitions), the type of LPS, its purity and the dispersity in the lipid chain structure, among others*.*

## Conclusions

In summary, we provide a detailed comparison of the self-assembly of a series of lipid A-based derivatives, considering a polydisperse bacterial-derived LPS as well as previously unexamined monodisperse synthetic derivatives. Distinct self-assembled nanostructures are observed *via* the powerful combination of SAXS and cryo-TEM. The observations are satisfactorily explained *via* a detailed analysis of the surfactant packing parameter. These compounds are of great current interest in vaccine formulation, and their self-assembly propensity may have an influence on bioactivity.

## Supporting Information Available

Parameters extracted from the fitting to the SAXS data, fluorescence and CD spectra, and additional cryo-TEM images (PDF)
