# Supplementary material for: Effect of Glycosylation on Self-Assembly of Lipid A Lipopolysaccharides in Aqueous Solutions
Source: Langmuir. 2023 Jun 8;39(24):8516–22. doi: 10.1021/acs.langmuir.3c00828 (PMC10286316; doi:10.1021/acs.langmuir.3c00828)
Supplement: Supplementary file 1 — la3c00828_si_001.pdf [file la3c00828_si_001.pdf]

Supporting Information

Effect of Glycosylation on Self-Assembly of Lipid A Lipopolysaccharides in Aqueous Solution

Valeria Castelletto,[1,*] Jani Seitsonen,[3] Ian W Hamley[1]

[1] *School of Chemistry, Food Biosciences and Pharmacy, University of Reading, Whiteknights, Reading RG6 6AD, U.K.*

[3] *Nanomicroscopy Center, Aalto University, Puumiehenkuja 2, FIN-02150 Espoo, Finland*

* v.castelletto@reading.ac.uk

Table S1. Parameters extracted from the fitting of the SAXS data in Figure 3a, using A form factor for a long cylindrical shell, together with that for a uniform sphere. Data was fitted using SASfit.[1]

	**0.1 wt% LPS[a]**	**0.5 wt% LPS**
N_1	0.06	0.40
R_c/ Å	19.3	19.3
σ/ Å	6.6	6.6
ΔR/ Å	20	20
η_c	1.0×10^{-6}	1.0×10^{-6}
η_s	-1.1×10^{-6}	-1.1×10^{-6}
N_2	0.028	0.16
R_o / Å	124	124
σ / Å	40	40
η	4.67×10^{-7}	4.67×10^{-7}
μ	-0.08	-0.08
BG	0.0006	0.0018

Key: Long Cylindrical Shell Form Factor: core radius with Gaussian polydispersity with height N_1, centre R_c (mean core radius) and width σ; shell thickness of headgroup, ΔR; scattering contrasts of core and shell η_c, η_s (η_{sol} = 0 was fixed); Length L = 500 Å fixed. **Uniform Sphere:** radius with Gaussian polydispersity with height N_2, radius R_o and width; scattering contrast η. **Background**, BG: constant.

Table S2. Parameters extracted from the fitting of the SAXS data in Figure 3b, using a form factor for a Gaussian bilayer nanosheet. Data was fitted using SASfit.[1]

	**0.1 wt% Kdo2L**	**0.5 wt% Kdo2L[a]**
N_1	1.0	5.0
t/ Å	42.0	42.0
Δt/ Å	0.1	0.45
η_{out} / cm^{-1}	4.58×10^{-5}	4.58×10^{-5}
σ_{out} / Å	5	5
η_{in} / cm^{-1}	-1.43×10^{-5}	-1.43×10^{-5}
σ_{in} / Å	5	5
D / Å	2000	2000
BG	0.00015	0.0003

Key: Gaussian bilayer: scale factor N_1, layer thickness t (Gaussian polydispersity Δt), scattering contrast of outer layer η_{out}, and inner layer η_{in}, Gaussian widths σ_{in} and σ_{out} of inner and outer layers respectively, D diameter (width) of nanosheets. **Background**, BG: constant.

[a] Parameters from fit for 0.1 wt% except scale factor N_1, Δt and BG.

Table S3. Parameters extracted from the fitting of the SAXS data in Figure 3c, using a form factor for a bilayer vesicle, together with a structure factor for thermally disordered layer structures. Data was fitted using SASfit.[1]

	**0.1 wt% MPLA[a]**	**0.5 wt% MPLA**
N	1	1.5
R_c/ Å	5	5
σ/ Å	5	5
t_h/ Å	14.5	14.5
t_t/ Å	13	13
η_h	6.16×10^{-7}	6.5×10^{-7}
η_t	-1.02×10^{-6}	-1.02×10^{-6}
N	2	2
d / Å	40	40
δ / Å	0.02	0.02
ν	1.5	1.5
BG	0.0009	0.0005

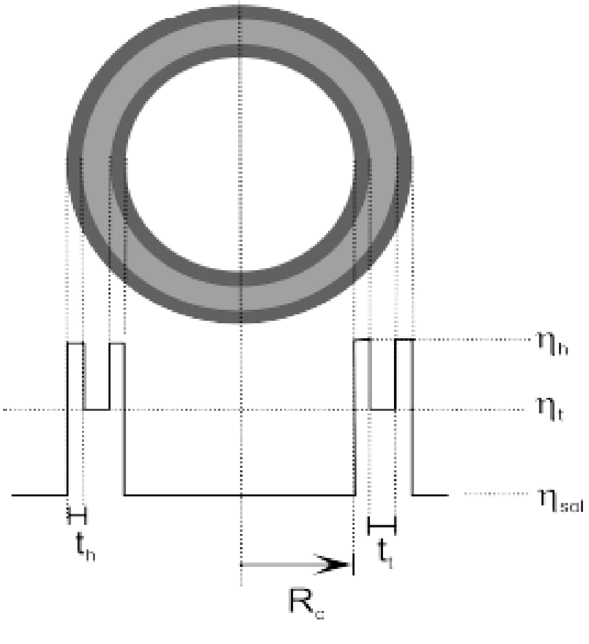

Key: Bilayer Vesicle Form Factor (see also schematic above): core radius with Gaussian polydispersity with height N, centre R_c (mean core radius) and width σ; thickness of headgroup, t_h; thickness of tail t_t; scattering contrasts of head and tail η_h, η_t (η_{sol} = 0 was fixed); **Thermally Disordered Lamellae Structure Factor:** number of layers, N; layer spacing d. Debye-Waller factor, δ; scaling constant for additional diffuse scattering term, ν. **Background**, BG: constant.

[a] Parameters from fit for 0.5 wt% except scale factors N_1, N_2 and BG.

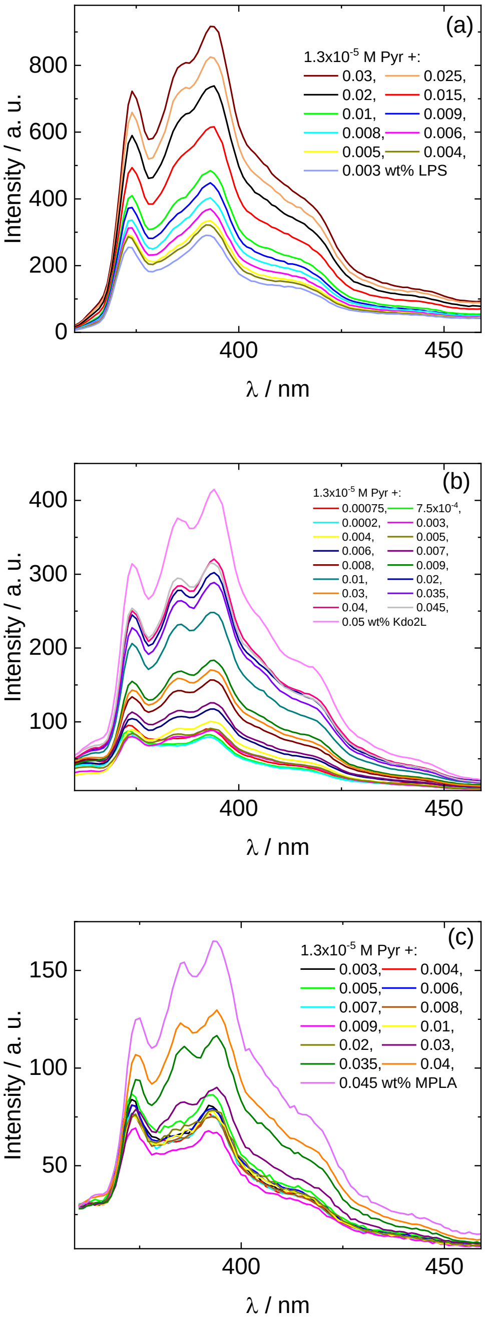

Figure S1. Fluorescence emission spectra for (a) LPS, (b) Kdo2L and (c) MPLA in pyrene solutions.

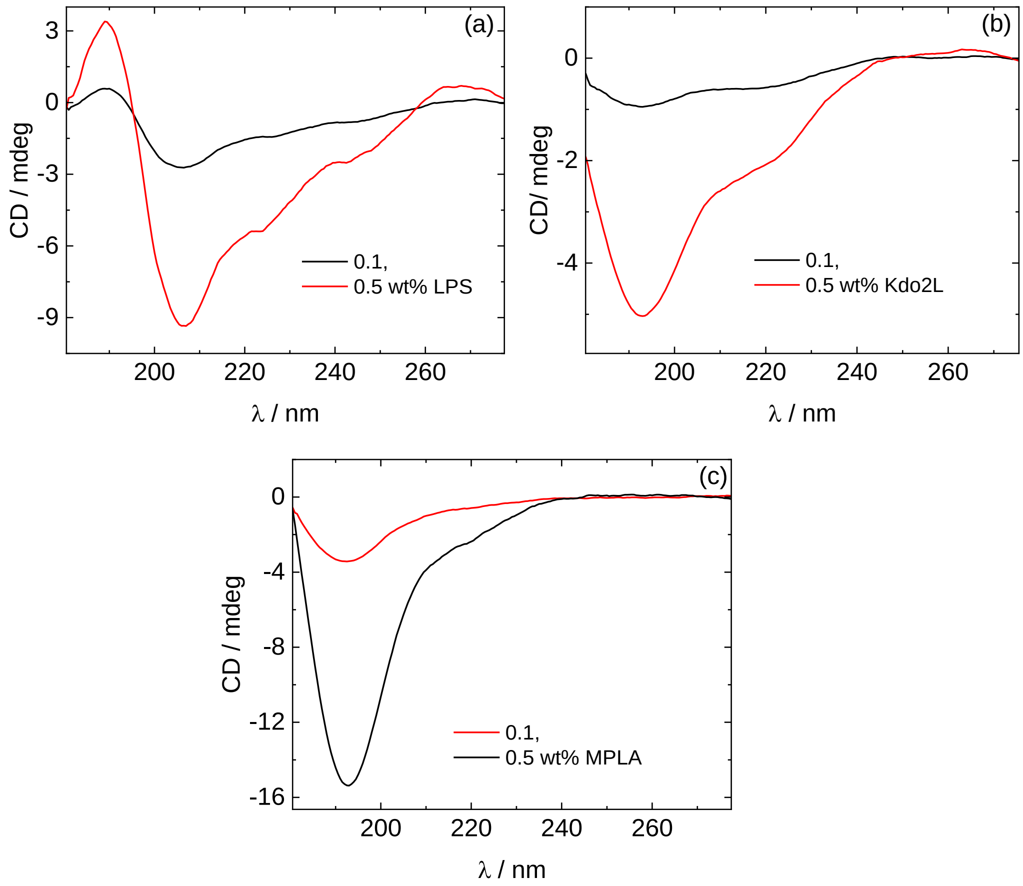

Figure S2. CD spectra for (a) LPS, (b) Kdo2L and (c) MPLA, at the concentrations shown.

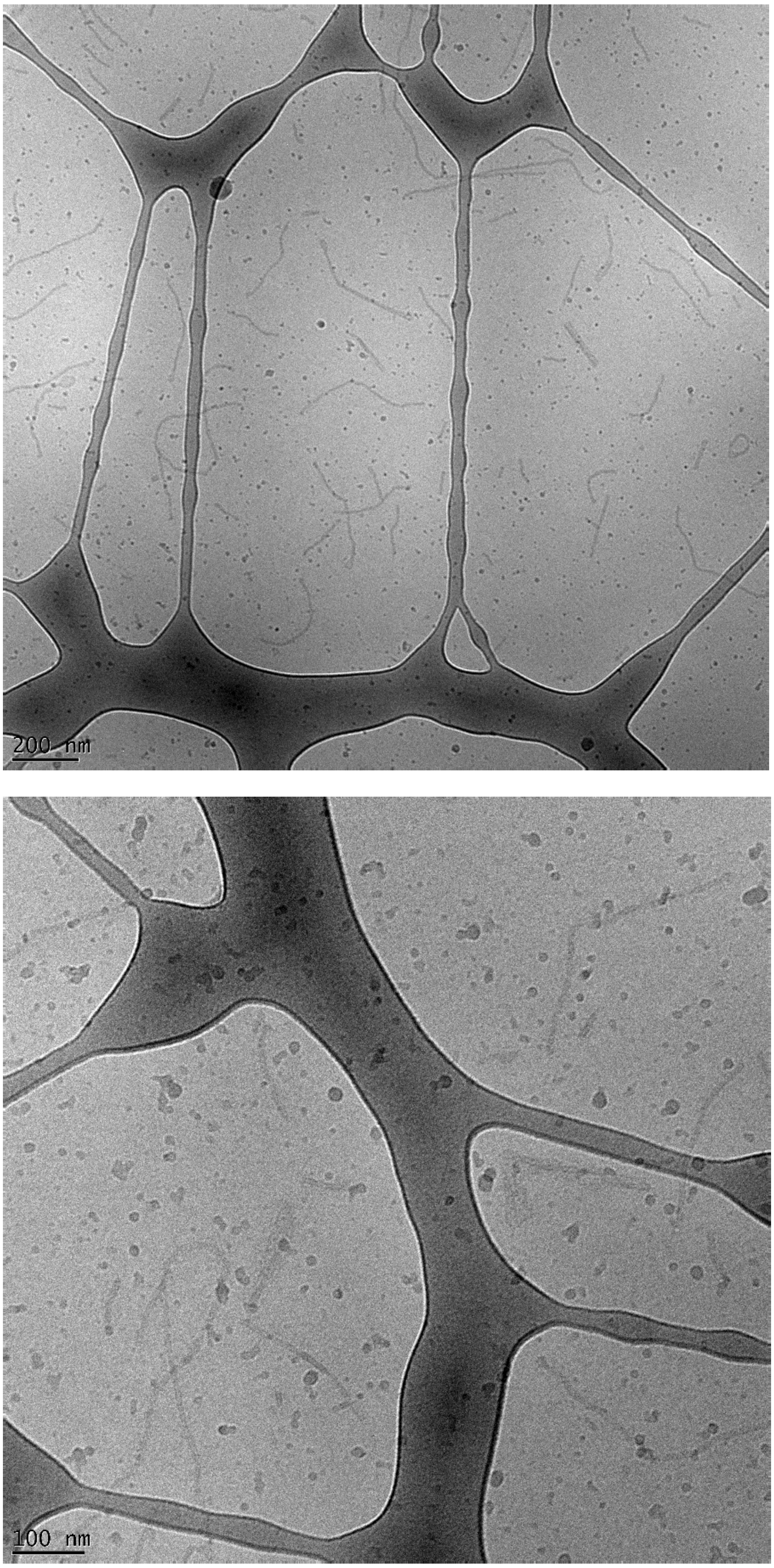

Figure S3. Representative additional cryo-TEM images for LPS (0.5 wt% aqueous solution).

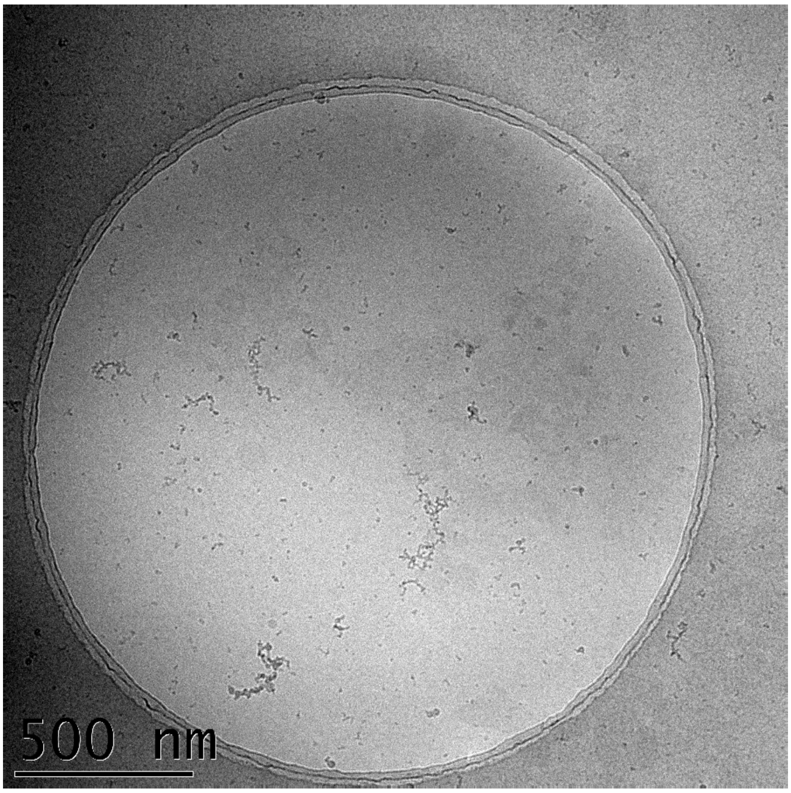

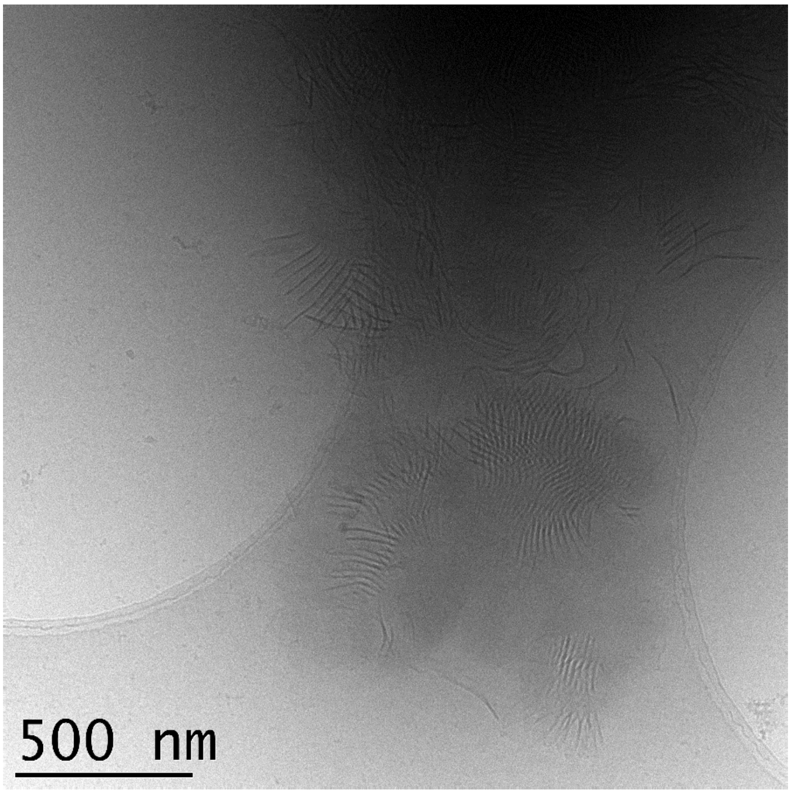

Figure S4. Representative additional cryo-TEM images for Kdo2L (0.5 wt% aqueous solution).

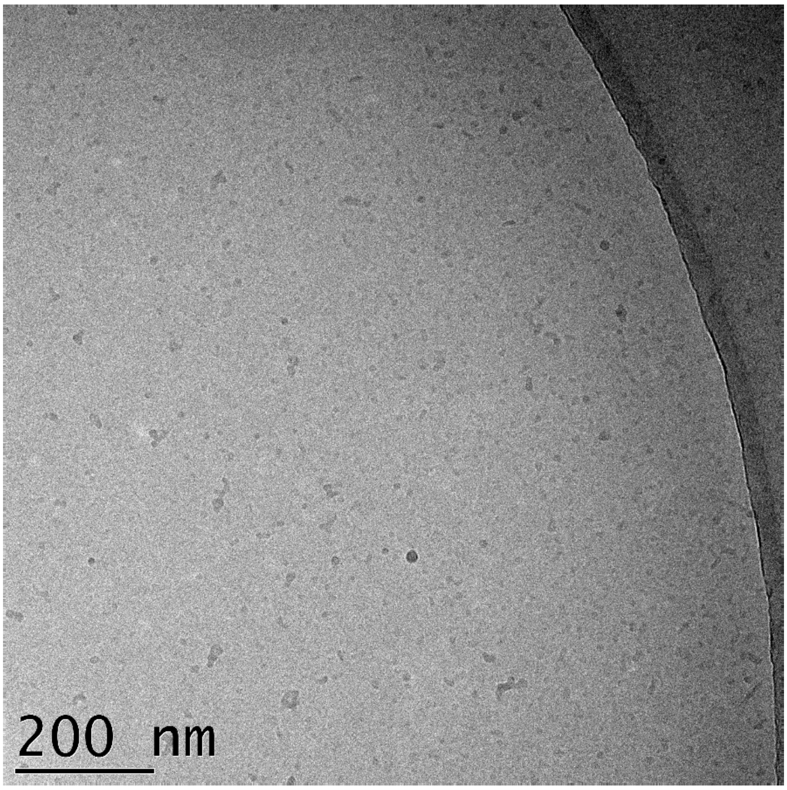

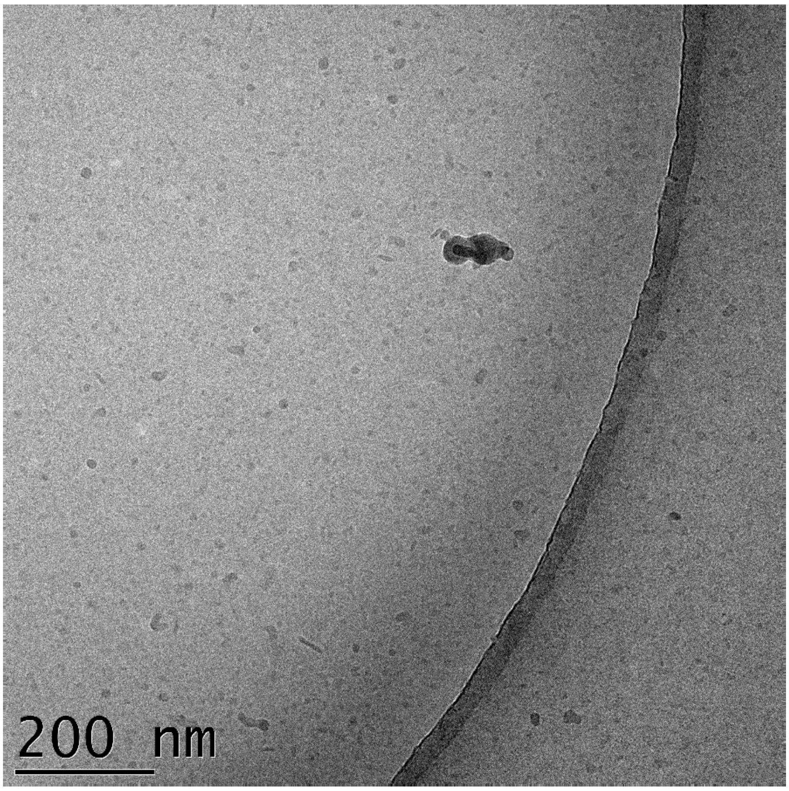

Figure S5. Representative additional cryo-TEM images for MPLA (0.5 wt% aqueous solution).

Reference

(1) Bressler, I.; Kohlbrecher, J.; Thünemann, A. F., SASfit: a tool for small-angle scattering data analysis using a library of analytical expressions. *J. Appl. Cryst*. **2015,** *48*, 1587-1598.
